# Meeting Report from the Second “Minimum Information for Biological and Biomedical Investigations” (MIBBI) workshop

**DOI:** 10.4056/sigs.147362

**Published:** 2010-12-25

**Authors:** Carsten Kettner, Dawn Field, Susanna-Assunta Sansone, Chris Taylor, Jan Aerts, Nigel Binns, Andrew Blake, Cedrik M. Britten, Ario de Marco, Jennifer Fostel, Pascale Gaudet, Alejandra González-Beltrán, Nigel Hardy, Jan Hellemans, Henning Hermjakob, Nick Juty, Jim Leebens-Mack, Eamonn Maguire, Steffen Neumann, Sandra Orchard, Helen Parkinson, William Piel, Shoba Ranganathan, Philippe Rocca-Serra, Annapaola Santarsiero, David Shotton, Peter Sterk, Andreas Untergasser, Patricia L. Whetzel

**Affiliations:** 1 Beilstein-Institut, Frankfurt am Main, Germany; 2 Centre for Ecology & Hydrology, Oxfordshire UK; 3 University of Oxford, Oxford e-Research Centre, Oxfordshire, UK; 4 The European Bioinformatics Institute (EBI), Wellcome Trust Genome Campus, Hinxton, Cambridgeshire, UK; 5 Faculty of Engineering - ESAT/SCD, Leuven University, Leuven-Heverlee, Belgium; 6 Division of Pathway Medicine, University of Edinburgh Medical School, Edinburgh, UK; 7 MRC Harwell, Harwell Science and Innovation Campus, Oxfordshire, UK; 8 Medical Department, University Medical Center, Johannes Gutenberg University-Mainz, Mainz, DE; 9 Consortium for Genomic Technology, Milano, Italy; 10 University of Nova Gorica, Nova Gorica, Slovenia; 11 US National Toxicology Program, NIEHS, NIH, NC, U.S.A.; 12 Swiss Institute of Bioinformatics, Geneva, CH; 13 Computational and Systems Medicine and Department of Computer Science, University College London, London, UK; 14 Department of Computer Science, Aberystwyth University, Aberystwyth, UK; 15 Center for Medical Genetics, Ghent University, Ghent, Belgium; 16 Department of Plant Biology, University of Georgia, Athens, GA, U.S.A.; 17 Department of Stress- and Developmental Biology, Institute for Plant Biochemistry, Halle, DE; 18 Peabody Museum of Natural History, Yale University, New Haven, CT, U.S.A.; 19 Macquarie University, Sydney NSW, Australia; 20 National University of Singapore, Singapore; 21 The Mario Negri Institute for Pharmacological Research, Cancer Pharmacology, 20156 Milan, Italy; 22 Image Bioinformatics Research Group, Department of Zoology, University of Oxford, Oxford, UK; 23 Wellcome Trust Sanger Institute, Wellcome Trust Genome Campus, Hinxton, Cambridgeshire, UK; 24 Zentrum für Molekulare Biologie der Universität Heidelberg, Heidelberg, Germany; 25 The National Center for Biomedical Ontology / Stanford Center for Biomedical Informatics Research, Stanford University, Stanford, CA, U.S.A.

## Abstract

This report summarizes the proceedings of the second workshop of the ‘Minimum Information for Biological and Biomedical Investigations’ (MIBBI) consortium held on Dec 1-2, 2010 in Rüdesheim, Germany through the sponsorship of the Beilstein-Institute. MIBBI is an umbrella organization uniting communities developing Minimum Information (MI) checklists to standardize the description of data sets, the workflows by which they were generated and the scientific context for the work. This workshop brought together representatives of more than twenty communities to present the status of their MI checklists and plans for future development. Shared challenges and solutions were identified and the role of MIBBI in MI checklist development was discussed. The meeting featured some thirty presentations, wide-ranging discussions and breakout groups. The top outcomes of the two-day workshop as defined by the participants were: 1) the chance to share best practices and to identify areas of synergy; 2) defining a series of tasks for updating the MIBBI Portal; 3) reemphasizing the need to maintain independent MI checklists for various communities while leveraging common terms and workflow elements contained in multiple checklists; and 4) revision of the concept of the MIBBI Foundry to focus on the creation of a core set of MIBBI modules intended for reuse by individual MI checklist projects while maintaining the integrity of each MI project. Further information about MIBBI and its range of activities can be found at http://mibbi.org/.

## Introduction

There is now a growing trend for grass-roots communities of researchers to define MI checklists for the description and contextualization of data, in order to facilitate sharing and to fully describe those data for publication and review. These guidelines are used both to describe data and the processes by which they were created, and to guide the creation of electronic resources for the storage of such descriptions. With the publication of a community-level paper in 2008 [1], the ‘Minimum Information for Biological and Biomedical Investigations’ (MIBBI) initiative was formally established, having begun in 2006 when a group of MI checklist project leaders came together to discuss the increase in common activity within the data sharing community and the consequent need for more efficient networking and collaboration where possible. MIBBI is intended to bring together community leaders for all MI checklist projects, to tackle shared challenges, develop best practice and create new solutions that ease the development of MI checklists and help to promote their implementation and adoption.

Seventeen representatives of minimum information checklist communities attended the first MIBBI meeting in 2008. Today some thirty-six community checklists are registered in the MIBBI Portal. This second MIBBI meeting once again convened the representatives of these minimum information checklist communities for a two day workshop in the historic Hotel Jagdschloss Niederwald near Rüdesheim, Germany on Dec 1-2, 2010. The setting and the limited number of participants provided a convivial atmosphere for the ready exchange of thoughts and ideas. The meeting was organized by Carsten Kettner (Beilstein-Institut), Chris Taylor (European Bioinformatics Institute), Susanna-Assunta Sansone (University of Oxford) and Dawn Field (Centre for Ecology and Hydrology) and was hosted and supported by the Beilstein-Institut.

The three primary objectives of the meeting were to: 1) review and advise on the current state of the MIBBI project; 2) formalize the ownership, upkeep and further development of MIBBI Foundry module content; and 3) reach a position on interactions with other standards projects and resources. The meeting included a series of community introductions, formal presentations on key topics to seed debate, extended discussion sessions and breakout groups.

## Community Introductions

The meeting opened with presentations by the checklist representatives present at the workshop. Attendees representing projects that have generated one or more MI specifications were requested to prepare a three-minute introduction to describe the scope and status of those specifications. Each presentation covered: 1) the scope of the specification(s) they represent; 2) the status of the specification(s) and any associated resources; and 3) a summary of their contributors and means of support. These slides, along with others from the meeting, are available *via* the MIBBI site (http://mibbi.org/). The checklists presented are listed in Table 1 along with a reference where one exists. They cover a wide range of data types and are in all stages of development from just-envisioned to well-established within the community.

**Table 1:** Checklists presented at the second MIBBI workshop^*^.

**Project Name**	**Presenter Name**	**Publication and website (where available)**
MIQAS	Jan Aerts (ESAT/SCD)	http://miqas.sourceforge.net/
MIARE	Nigel Binns (Edinburgh)	http://www.miare.org/
		
		
		
		
		
MIMPP	Andy Blake (MRC Harwell)	[2], http://www.interphenome.org/
		
		
		
		
		
MIATA	Cedrik M. Britten (Mainz)	[3,4], http://www.mitataproject.org/
		
		
		
		
		
MIPFE	Ario de Marco (IFOM-IEO)	[5], http://mipfe.org/
		
		
		
		
		
TBC	Jennifer Fostel (NIEHS)	[6]
		
		
		
		
		
MINSEQE MIAME	Jennifer Fostel (NTP, HESI) on behalfof Helen Parkinson (EBI, UK)	http://www.mged.org/minseqe/[7], http://www.mged.org/Workgroups/MIAME/miame.html
		
		
		
		
		
BIODBCORE	Pascale Gaudet (ISB)	[8], http://biocurator.org/biodbcore.shtml
		
		
		
		
		
GIATE	Alejandra González-Beltrán (UCL)	[9-11], http://www.antibodysociety.org/data/datastandards.php
		
		
		
		
		
CIMR	Nigel Hardy (Aberystwyth)	[12], http://msi-workgroups.sourceforge.net/
		
		
		
		
		
MIQE / MIqPCR	Jan Hellemans (Gent) Andreas Untergasser (Heidelberg)	[13-15], http://www.rdml.org/miqe.php
		
		
		
		
		
MIRIAM MIASE	Nick Juty (EBI)	[16], http://biomodels.net/miriam http://biomodels.net/miase/
		
		
		
		
		
STRENDA	Carsten Kettner (Beilstein-Institut)	[17], http://www.strenda.org/
		
		
		
		
		
MIAPA	Jim Leebens-Mack (Georgia)	[18]
		
		
		
		
		
MIMIX MIAPAR MIABE	Henning Hermjaokb (EBI)	[19], http://www.psidev.info/ [20], http://www.psidev.info/ http://www.psidev.info/
		
		
		
		
		
MIABi	Shoba Ranganathan (Macquarie, NUS)	[21]
		
		
		
		
		
MIIDI	David Shotton (Oxford)	http://imageweb.zoo.ox.ac.uk/wiki/index.php/MIIDI
		
		
		
		
		
MIGS/MIMS/MIENS	Peter Sterk (Sanger)	[22,23], http://gensc.org/gc_wiki/index.php/MIGS/MIMS/MIENS
		
		
		
**Additional projects** MIACA (http://miaca.sourceforge.net/); MIAME/Env [24]; MIAME/Nutr & MIAME/Tox (http://www.mged.org/Workgroups/rsbi/rsbidetail.html#rationale); MIAME/Plant [25]; MIAPE [26]; MIASPPE [27]; MIEME [28]; MIFlowCyt [29]; MIfMRI [30]; MIGen (http://migen.sourceforge.net/); MINI [31]; MINIMESS [32]; MISFISHIE [33]		

^*^ The checklist’s acronym, the presenter and primary publication (where one exists) or web link are given. The final row lists those projects registered with MIBBI that could not be presented during the meeting.

## The MIBBI Foundry - the working intersection of MI checklist groups

The next session reviewed the concept and status of the MIBBI Foundry. To set the stage, Chris Taylor gave an overview of the MIBBI Foundry and reported on progress towards a list of ‘MIBBI modules’. The primary purpose of the MIBBI Foundry is to coordinate checklist communities’ efforts to regularize the content of their reporting requirements through joint working where their respective scopes overlap. The MIBBI paper [1] presented the degree of overlap between checklists registered with MIBBI at the time of publication in tabular form, highlighting that certain concepts are frequently repeated across checklists while others are unique. The repetition of concepts across checklists suggested that it might be possible to modularize the parts of those checklists to provide reusable components to checklist projects to minimize the arbitrary differences in the reporting of common concepts that would otherwise naturally occur. To date, thirty-six MIBBI modules have been derived by merging and adapting parts of original community checklists. These draft modules are available on the MIBBI site. The presentation closed with a call for wider participation in the Foundry project.

The participants then discussed the merits of a modular approach to checklist development and use. All agreed that it was sensible for checklist communities to work together both to promote best practice and to ensure as far as possible that their checklists are orthogonal (non-overlapping) and semantically comparable; for example, by tagging individual checklist items with ontology terms.

Breakout groups were organized to tackle a variety of issues surrounding the development, maintenance and use of MI checklists and to deliberate on a variety of questions about the MIBBI Foundry. There were three groups, designed to reflect the interests of ‘wet lab’ bench scientists, bioinformaticians/developers and the ‘meta-standards’ community (*i.e.*, those seeking to integrate standards-based resources). These groups were led by Carsten Kettner (Beilstein-Institut), Pascale Gaudet (Swiss Institute of Bioinformatics) and Henning Hermjakob (European Bioinformatics Institute) respectively, with Jan Hellemans (University of Gent), Jim Leebens-Mack (University of Georgia), and Trish Whetzel (Stanford University) reporting back on their discussions. The groups ranged wide, but common themes emerged, as summarized at the end of this report.

### Towards a MIBBI Core - a shared resource for community checklist developers

Common themes emerged from individual MI group presentations including a desire for flexible and extensible tools for the facilitation of data sharing. In response, Philippe Rocca-Serra (University of Oxford) presented the ISA infrastructure, a data sharing software suite that depends critically on the dissemination of mature checklists for the capture of standards-compliant experimental metadata. The ISA infrastructure [34] provides a set of tools dedicated to the annotation and reporting of classic and functional genomics experiments, enabling efficient reporting, semantic tagging and support for submission to various public databases. The highly configurable nature of the architecture has been applied to implement a number of MI checklists. Preliminary validation work resulted in the creation of configurations for MIENS, MISFISHIE, MIAME and MIFlowCyt in direct collaboration with representatives of those checklists; the first components of a library of configurations for those and many other MI checklists. For groups defining minimal annotation that lack the resource to develop syntax, editing and validation tools, the ISA infrastructure was presented as providing a considerable head start for implementing and delivering annotation solutions quickly and effectively.

There then followed a presentation on MICheckout, developed by Chris Taylor and Eamonn Maguire (University of Oxford). This tool, built with Adobe Flash, Java, RESTful web services, XML and XSLT, allows users to compile MIBBI Foundry modules into custom lists that can be viewed or downloaded in various formats. The initial version of the tool, as presented at the workshop, provides users with access to the full range of modules in the MIBBI Foundry; those modules having been derived from the checklists registered in the MIBBI Portal. The modules are stored in XML, which is transformed by the tool on demand to provide the requested output. Subsequent discussion centered on the healthy tension that exists between the desire for integration where MI checklists overlap and the tailoring of checklists to meet the needs of particular user communities. In its current form, MICheckout was considered useful for developers, and for end-users employing multiple technologies. However, scientists, and reviewers for funders, journals or regulators require a much-simplified interface that leans heavily on the domain/technique-specific checklists listed in the MIBBI Portal to ensure that equivalent users obtain identical checklists built on best practices within the field.

The focus then shifted to MIBBI in the wider data sharing landscape, including an overview of the new BioSharing initiative by Susanna Sansone. The proliferation of standardization efforts (not just checklists, but also terminologies and exchange formats) is a positive indicator of stakeholder engagement, but brings with it new sociological and technological challenges — creating interoperability and avoiding the overlaps and duplication of effort that hamper widespread uptake and hinder the development of data sharing tools. The BioSharing (http://www.biosharing.org) initiative [35,36], which includes the MIBBI community, aims to address this challenge, working at the global level to build stable links between funders, well-constituted standards projects, journals and other stakeholders to expedite communication and the production of an integrated standards-based framework for the capture and sharing of high-throughput genomics and functional genomics data. For example, the International Society for Biocuration, a contributor to the work of the BioSharing project, has recently produced the MIBBI-registered BioDBCore checklist [8], which aims to improve standardization across biological databases. Under development with Annapaola Santarsiero (The Mario Negri Institute) are a ‘one-stop shop’ catalogue and a communication forum to help centralize information on bioscience data policies, standards and links to other related portals, including MIBBI and the National Center for Biomedical Ontology (NCBO) BioPortal, and to establish the formal communication channels needed to maintain links between stakeholders.

Trish Whetzel (National Center for Biomedical Ontology) then gave a brief overview of the NCBO, which provides online tools and a Web portal - the BioPortal [26] - for biomedical researchers, enabling them to access, review and integrate disparate ontological resources covering all aspects of biomedical investigation and clinical practice. A major focus of NCBO is the use of biomedical ontologies to aid in the management and analysis of data; here the interplay with MI checklists is pivotal.

Returning to their working groups, the participants next addressed two subjects: the general direction of MIBBI (*i.e.*, how the project should evolve); and the design of the MICheckout tool. Again, the outcomes of these discussions have been fed into the summary at the end of this report.

### Wrap up session and outcomes

The group then spent one hour wrapping up and deciding on actions in a session chaired by Dawn Field, who gave a brief overview of the meeting and then opened the floor for discussion. All were in agreement that: 1) MIBBI should continue; 2) future meetings would be of value; 3) the Portal should be improved; 4) that there is a need to strike a balance between the independence of MI communities (as listed in the Portal) and the need for harmonization (through the Foundry); 5) that communication should be improved; and that 6) in general there are many ways in which the project could develop.

**Attendees felt that the most positive outcomes of the meeting were:**
- The chance to meet other MI checklist project coordinators and exchange knowledge.
- The clarification of the roles of MIBBI:
  o For developers: promoting best practice on creating MIs; working with those seeking validation for their implementation (*e.g.*, software/instrument vendors); supporting DOIs (http://datacite.org/) and ORCIDs (http://orcid.org/); providing ‘common’ checklist modules that can be re-used by communities to improve compatibility; development and maintenance of mappings between community checklists; providing tutorials and forums for discussion.
  o for end-users: provision of guidance and tools to simplify the identification of relevant standards; links to summaries of journals’ and funders’ requirements and tools to support those requirements; provision of instructive examples of usage; and the development of a wizard to help end-users identify checklists with which they should be trying to comply.
- The agreement to provide a core set of MIBBI modules covering common bioscience workflow components for reuse in communities’ own checklists.
- The identification of tools to help users to meet the requirements of several MI checklists, such as ISA software suite.
- The commitment to work together to help promote the efforts of each group providing checklists (for example, by working towards a journal special issue).

**Agreements on the specific roles and further development of the various parts of MIBBI included:**
1. Portal
  Roles: maintain up-to-date versions of descriptions of MI projects; actively engage with checklist project representatives, for example, through regular email circulars.
  Best practices: ensure all projects provide standard descriptions of their MI checklists; define ‘style guidance’ for checklists (which currently vary greatly); support DOIs and ORCIDs; recommend the use of version control and the timely handling of requests for revisions.
  Certification/uptake: creation of a process to certify tools/systems, to maximize adoption (because the more tools that are genuinely compliant, the easier it is for users to comply); opening of dialogs with national standards groups.
2. Foundry
  Role: enhance the compatibility of community checklists without encroaching on their independence.
  Common elements: the MIBBI Foundry should offer a core set of modules covering common areas of workflows; those modules and their components should be semantically tagged and available in several formats, to be reused by individual communities to enhance checklist compatibility.
  Project-specific content: offer the facility to host or mirror communities’ checklist content to facilitate its discovery and use, ensuring that users have straightforward access to up-to-date guidance.
3. Website
  Role: to provide information about the project to newcomers and others in concise, bulleted form.
  User management: the website should cater to developers and other kinds of user (bench scientists, reviewers of various kinds) separately, acknowledging their different needs.
4. MIBBI in the context of the BioSharing initiative
  Role: to represent the interests of the MI checklist community and to ensure stable links to other standards and the bodies that represent them.

## References

[1] Taylor CF Field D Sansone SA Aerts J Apweiler R Ashburner M Ball CA Binz PA Bogue M Booth T Promoting coherent minimum reporting guidelines for biological and biomedical investigations: the MIBBI project. Nat Biotechnol 2008; 26:889-896 10.1038/nbt.1411 18688244 PMC2771753

[2] Hancock JM Adams NC Aidinis V Blake A Bogue M Brown SD Chesler EJ Davidson D Duran C Eppig JT Mouse Phenotype Database Integration Consortium: integration [corrected] of mouse phenome data resources. Mamm Genome 2007; 18:157-163 17436037 10.1007/s00335-007-9004-x PMC4230762

[3] Janetzki S Britten CM Kalos M Levitsky HI Maecker HT Melief CJ Old LJ Romero P Hoos A Davis MM MIATA"-minimal information about T cell assays. Immunity 2009; 31:527-528 10.1016/j.immuni.2009.09.007 19833080 PMC3762500

[4] Britten CM Janetzki S Van der Burg SH Huber C Kalos M Levitsky HI Maecker HT Melief CJ O'Donnell-Tormey J Old LJ Minimal information about T cell assays: the process of reaching the community of T cell immunologists in cancer and beyond. Cancer Immunol Immunother 2010; (Nov):16 21080166 10.1007/s00262-010-0940-z PMC3029829

[5] de Marco A Minimal information: an urgent need to assess the functional reliability of recombinant proteins used in biological experiments. Microb Cell Fact 2008; 7:20 10.1186/1475-2859-7-20 18647423 PMC2499994

[6] Fostel JM Burgoon L Zwickl C Lord P Corton JC Bushel PR Cunningham M Fan L Edwards SW Hester S Toward a checklist for exchange and interpretation of data from a toxicology study. Toxicol Sci 2007; 99:26-34 10.1093/toxsci/kfm090 17442663

[7] Brazma A Hingamp P Quackenbush J Sherlock GSP Stoeckert C Aach J Ansorge W Ball CA Causton HC Gaasterland T Minimum information about a microarray experiment (MIAME)-toward standards for microarray data. Nat Genet 2001; 29:365-371 10.1038/ng1201-365 11726920

[8] Gaudet P, Bairoch A, Field D, Sansone SA, Taylor C, Attwood TK, Bateman A, Blake JA, Bult CJ, Cherry JM, e*t al* Towards BioDBcore: a community-defined information specification for biological databases. *Nucleic Acids Res* 2010. 10.1093/nar/gkq1173 PMC3013734 21097465

[9] Yong M Begent R Best use of experimental data in cancer informatics. Future Oncol 2010; 6:1551-1562 10.2217/fon.10.108 21062155

[10] Yong M Tolner B Nagl S Pedley RB Chester K Green AJ Mayer A Sharma S Begent R Data standards for minimum information collection for antibody therapy experiments. Protein Eng Des Sel 2008; 22:221-224 10.1093/protein/gzp003 19224941

[11] Gonzalez-Beltran A, Yong M, Begent R. Towards an unambiguous and formal description of cancer therapy experiments. In “Building a Collaborative Biomedical Network“, caBIG Annual Meeting, September 13-15, 2010, Washington, D.C., U.S.A. 2009.

[12] Sansone SA Fan T Goodacre R Griffin JL Hardy NW Kaddurah-Daouk R Kristal BS Lindon J Mendes P Morrison N The metabolomics standards initiative. Nat Biotechnol 2007; 25:846-848 10.1038/nbt0807-846b 17687353

[13] Bustin SA Benes V Garson JA Hellemans J Huggett J Kubista M Mueller R Nolan T Pfaffl MW Shipley GL The MIQE guidelines: minimum information for publication of quantitative real-time PCR experiments. Clin Chem 2009; 55:611-622 10.1373/clinchem.2008.112797 19246619

[14] Bustin SA Beaulieu JF Huggett J Jaggi R Kibenge FS Olsvik PA Penning LC Toegel S MIQE precis: Practical implementation of minimum standard guidelines for fluorescence-based quantitative real-time PCR experiments. BMC Mol Biol 2010; 11:74 10.1186/1471-2199-11-74 20858237 PMC2955025

[15] Lefever S Hellemans J Pattyn F Przybylski DR Taylor C Geurts R Untergasser A Vandesompele J RDML: structured language and reporting guidelines for real-time quantitative PCR data. Nucleic Acids Res 2009; 37:2065-2069 10.1093/nar/gkp056 19223324 PMC2673419

[16] Novère N Finney A Hucka M Bhalla US Campagne F Collado-Vides J Crampin EJ Halstead M Klipp E Mendes P Minimum information requested in the annotation of biochemical models (MIRIAM). Nat Biotechnol 2005; 23:1509-1515 10.1038/nbt1156 16333295

[17] Apweiler R Armstrong R Bairoch A Cornish-Bowden A Halling PJ Hofmeyr JH Kettner C Leyh TS Rohwer J Schomburg D A large-scale protein-function database. Nat Chem Biol 2010; 6:785 10.1038/nchembio.460 20956966 PMC3245624

[18] Leebens-Mack J Vision T Brenner E Bowers JE Cannon S Clement MJ Cunningham CW dePamphilis C deSalle R Doyle JJ Taking the first steps towards a standard for reporting on phylogenies: Minimum Information About a Phylogenetic Analysis (MIAPA). OMICS 2006; 10:231-237 10.1089/omi.2006.10.231 16901231 PMC3167193

[19] Orchard S Salwinski L Kerrien S Montecchi-Palazzi L Oesterheld M Stumpflen V Ceol A Chatr-aryamontri A Armstrong J Woollard P The minimum information required for reporting a molecular interaction experiment (MIMIx). Nat Biotechnol 2007; 25:894-898 10.1038/nbt1324 17687370

[20] Bourbeillon J Orchard S Benhar I Borrebaeck C de Daruvar A Dubel S Frank R Gibson F Gloriam D Haslam N Minimum information about a protein affinity reagent (MIAPAR). Nat Biotechnol 2010; 28:650-653 10.1038/nbt0710-650 20622827

[21] Tan TW Tong JC De Silva M Lim KS Ranganathan S Advancing Standards for Bioinformatics Activities: Persistence, Reproducibility, Disambiguation and Minimum Information about a Bioinformatics Investigation (MIABi). BMC Genomics 2010; 11(Suppl 4):S27 10.1186/1471-2164-11-S4-S27 21143811 PMC3005918

[22] Field D Garrity G Gray T Morrison N Selengut J Sterk P Tatusova T Thomson N Allen MJ Angiuoli SV The minimum information about a genome sequence (MIGS) specification. Nat Biotechnol 2008; 26:541-547 10.1038/nbt1360 18464787 PMC2409278

[23] Yilmaz P, Kottmann R, Field D, Knight R, Cole JR, Amaral-Zettler L, Gilbert JA, Karsch-Mizrachi I, Johnston A, Cochrane G, *et al* The “Minimum Information about an ENvironmental Sequence” (MIENS) specification. Available from Nature Precedings <http://dx.doi.org/10.1038/npre.2010.5252.2> 2010.

[24] Morrison N Wood AJ Hancock D Shah S Hakes L Gray T Tiwari B Kille P Cossins A Hegarty M Annotation of environmental OMICS data: application to the transcriptomics domain. OMICS 2006; 10:172-178 10.1089/omi.2006.10.172 16901223

[25] Zimmermann P Schildknecht B Craigon D Garcia-Hernandez M Gruissem W May S Mukherjee G Parkinson H Rhee S Wagner U MIAME/Plant - adding value to plant microarrray experiments. Plant Methods 2006; 2:1 10.1186/1746-4811-2-1 16401339 PMC1334190

[26] Noy NF Shah NH Whetzel PL Dai B Dorf M Griffith N Jonquet C Rubin DL Storey MA Chute CG BioPortal: ontologies and integrated data resources at the click of a mouse. Nucleic Acids Res 2009; 37:W170-W173 10.1093/nar/gkp440 19483092 PMC2703982

[27] Carrascal M Ovelleiro D Casas V Gay M Abian J Phosphorylation analysis of primary human T lymphocytes using sequential IMAC and titanium oxide enrichment. J Proteome Res 2008; 7:5167-5176 10.1021/pr800500r 19367720

[28] Frishkoff GW, LePendu P, Frank RM, Liu H, Dou D. Development of Neural Electromagnetic Ontologies (NEMO): Ontology-based Tools for Representation and Integration of Event-related Brain Potentials. Available from Nature Precedings <http://dx.doi.org/doi:10.1038/npre.2009.3458.1> 2009.

[29] Lee JA Spidlen J Boyce K Cai J Crosbie N Dalphin M Furlong J Gasparetto M Goldberg M Goralczyk EM MIFlowCyt: the minimum information about a Flow Cytometry Experiment. Cytometry A 2008; 73A:926-930 10.1002/cyto.a.20623 18752282 PMC2773297

[30] Poldrack RA Fletcher PC Henson RN Worsley KJ Brett M Nichols TE Guidelines for reporting an fMRI study. Neuroimage 2008; 40:409-414 10.1016/j.neuroimage.2007.11.048 18191585 PMC2287206

[31] Gibson F, Overton PG, Smulders TV, Schultz SR, Eglen SJ, Ingram CD, Panzeri S, Bream P, Whittington M, Sernagor E, *et al* Minimum Information about a Neuroscience Investigation (MINI): Electrophysiology. Available from Nature Precedings 2009.

[32] Raes J Foerstner KU Bork P Get the most out of your metagenome: computational analysis of environmental sequence data. Curr Opin Microbiol 2007; 10:490-498 10.1016/j.mib.2007.09.001 17936679

[33] Deutsch EW Ball CA Berman JJ Bova GS Brazma A Bumgarner RE Campbell D Causton HC Christiansen JH Daian F Minimum information specification for in situ hybridization and immunohistochemistry experiments (MISFISHIE). Nat Biotechnol 2008; 26:305-312 10.1038/nbt1391 18327244 PMC4367930

[34] Rocca-Serra P Brandizi M Maguire E Sklyar N Taylor C Begley K Field D Harris S Hide W Hofmann O ISA software suite: supporting standards-compliant experimental annotation and enabling curation at the community level. Bioinformatics 2010; 26:2354-2356 10.1093/bioinformatics/btq415 20679334 PMC2935443

[35] Field D Sansone SA Collis A Booth T Dukes P Gregurick SK Kennedy KL Kolar P Kolker E Maxon M 'Omics Data Sharing. Science 2009; 326:234-236 10.1126/science.1180598 19815759 PMC2770171

[36] Field D, Sansone SA, DeLong EF, Sterk P, Friedberg I, Gaudet P, Lewis S, Kottmann R, Hirschman L, Garrity G, *et al* Meeting Report: BioSharing at ISMB 2010. *Stand. Genomics Sci* 2010;**3**:1-9. 10.4056/sigs/1403501 PMC3035313 21304729

